# Role of Inflammation in Short Sleep Duration Across Childhood and Psychosis in Young Adulthood

**DOI:** 10.1001/jamapsychiatry.2024.0796

**Published:** 2024-05-08

**Authors:** Isabel Morales-Muñoz, Steven Marwaha, Rachel Upthegrove, Vanessa Cropley

**Affiliations:** 1 Institute for Mental Health, School of Psychology, University of Birmingham, Birmingham, United Kingdom; 2 Specialist Mood Disorders Clinic, Zinnia Centre, Birmingham, United Kingdom; 3 The Barberry National Centre for Mental Health, Birmingham, United Kingdom; 4 Early Intervention Service, Birmingham Women’s and Children’s NHS Trust, Birmingham, United Kingdom; 5 Melbourne Neuropsychiatry Centre, Department of Psychiatry, The University of Melbourne & Melbourne Health, Melbourne, Victoria, Australia

## Abstract

**Importance:**

Short sleep duration over a prolonged period in childhood could have a detrimental impact on long-term mental health, including the development of psychosis. Further, potential underlying mechanisms of these associations remain unknown.

**Objective:**

To examine the association between persistent shorter nighttime sleep duration throughout childhood with psychotic experiences (PEs) and/or psychotic disorder (PD) at age 24 years and whether inflammatory markers (C-reactive protein [CRP] and interleukin 6 [IL-6]) potentially mediate any association.

**Design, Setting, and Participants:**

This cohort study used data from the Avon Longitudinal Study of Parents and Children. Data analysis was conducted from January 30 to August 1, 2023.

**Exposures:**

Nighttime sleep duration was collected at 6, 18, and 30 months and at 3.5, 4 to 5, 5 to 6, and 6 to 7 years.

**Main Outcomes and Measures:**

PEs and PD were assessed at age 24 years from the Psychosislike Symptoms Interview. CRP level at ages 9 and 15 years and IL-6 level at 9 years were used as mediators. Latent class growth analyses (LCGAs) were applied to detect trajectories of nighttime sleep duration, and logistic regressions were applied for the longitudinal associations between trajectories of nighttime sleep duration and psychotic outcomes at 24 years. Path analyses were applied to test CRP and IL-6 as potential mediators.

**Results:**

Data were available on 12 394 children (6254 female [50.5%]) for the LCGA and on 3962 young adults (2429 female [61.3%]) for the logistic regression and path analyses. The LCGA identified a group of individuals with persistent shorter nighttime sleep duration across childhood. These individuals were more likely to develop PD (odds ratio [OR], 2.50; 95% CI, 1.51-4.15; *P* < .001) and PEs (OR, 3.64; 95% CI, 2.23-5.95; *P* < .001) at age 24 years. Increased levels of IL-6 at 9 years, but not CRP at 9 or 15 years, partially mediated the associations between persistent shorter sleep duration and PD (bias-corrected estimate = 0.003; 95% CI, 0.002-0.005; *P* = .007) and PEs (bias-corrected estimate = 0.002; 95% CI, 0-0.003; *P* = .03) in young adulthood.

**Conclusions and Relevance:**

Findings of this cohort study highlight the necessity of addressing short sleep duration in children, as persistence of this sleep problem was associated with subsequent psychosis. This study also provides preliminary evidence for future targeted interventions in children addressing both sleep and inflammatory responses.

## Key Points

**Question:**

What is the association of persistent shorter sleep duration across childhood with psychosis in young adulthood?

**Findings:**

In this cohort study including 12 394 children and 3962 young adults, a group of individuals characterized by persistent shorter sleep duration from infancy until childhood were identified, and this group was significantly associated with psychosis at age 24 years. Further, elevated interleukin 6 (IL-6) levels at 9 years partially mediated these associations.

**Meaning:**

Shorter sleep duration across childhood, particularly if sustained at all time points, may be considered as a risk for the development of psychosis in adulthood; inflammation as measured by IL-6 level could be one of the potential mechanistic pathways.

## Teaser

This cohort study investigates the association between persistent shorter sleep duration across childhood with psychosis in young adulthood.

## Introduction

Short sleep duration can have a detrimental impact on a child’s development in the short, medium, and long term. For instance, children who do not get enough sleep are at increased risk for mental health problems,^1^ attention-deficit/hyperactivity disorder (ADHD),^2^ and/or behavioral problems.^3^

When considering the impact of short sleep duration in childhood, most studies have considered specific and isolated time points. However, as sleep duration is highly variable across childhood,^4^ this approach may not be very accurate to capture children with persistent sleep problems and, thus, at highest risk for adverse outcomes.^5^ For instance, children with lack of sleep at a specific time point may not experience this lack of sleep at later ages due to developmental changes^6^ or to improvement of environmental factors impacting sleep.^7^ Therefore, it is crucial to identify those children with persistent lack of sleep over time, as this is the group of individuals for whom the detrimental impact of short sleep will be greatest and who will experience the most negative outcomes in the long term.

Among mental health problems associated with short sleep duration is psychosis, which is a pervasive and distressing mental health condition.^8^ Sleep deprivation or sleep restriction studies support the potential causal role of short sleep duration in the development of psychotic symptoms^9,10,11^; however, these studies assessed sleep loss over the short term and cannot be used to understand its long-term impact. Longitudinal studies that investigate the effect of childhood sleep problems as a risk factor for psychosis are scarce and inconclusive, with most studies focusing on other sleep aspects, such as parasomnias.^12,13^ We recently examined the associations of behavioral sleep aspects in childhood with psychotic and borderline personality disorder (BPD) symptoms in adolescence and found that shorter sleep duration was associated with BPD but not with psychosis.^14^ However, this study selected a specific and isolated time point in childhood. It therefore remains possible that the chronicity of short sleep over the childhood period could constitute a risk factor for psychosis, rather than short sleep duration at a discrete developmental time point.

Potential mechanisms linking short sleep duration in childhood with later psychosis are likewise unexplored. Among potential candidates including neuromaturation, cognition, and biological mechanisms,^15^ inflammation has recently been suggested as a potential mediating factor,^16^ given known reciprocal links between both sleep and the immune system^17,18^ and evidence for low-grade inflammation in people with psychosis.^19^ For instance, existing evidence in adults suggests that short sleep duration prospectively predicts increases in inflammation,^20^ which would support a potential underlying role of inflammation in the associations between short sleep and psychosis. Nevertheless, to date, only 1 cross-sectional study has explored relationships between inflammatory markers, sleep, and psychosis,^21^ finding that outpatients with schizophrenia had worse sleep quality and higher levels of inflammatory markers compared with controls.

The current study examined the prospective association of persistent shorter sleep duration across childhood with psychosis in young adulthood, and the potential mediating role of inflammation. We focused on psychosis in young adulthood as this is when most cases of psychosis begin.^22^ We hypothesized that we would detect a small group of individuals with persistent shorter sleep duration across childhood, based on recent research on sleep duration trajectories across childhood.^23,24^ Further, persistent shorter sleep duration across childhood would increase the risk for psychosis,^10^ and elevated inflammatory markers would partially mediate these prospective associations.^16^

## Methods

### Participants

The Avon Longitudinal Study of Parents and Children (ALSPAC) is a UK birth cohort study examining the determinants of development, health, and disease during childhood and beyond.^25,26^ Pregnant women who were residents in Avon, UK, with expected dates of delivery between April 1, 1991, and December 31, 1992, were invited to take part. The initial number of pregnancies enrolled was 14 541. Of these, there were a total of 14 676 fetuses, resulting in 14 062 live births and 13 988 children alive at 1 year of age. Further details of the ALSPAC study are provided in eAppendix 1 in Supplement 1. Ethical approval was obtained from the law and ethics committee of the ALSPAC study. Written informed consent was obtained from the parents of the children. We followed the Strengthening the Reporting of Observational Studies in Epidemiology (STROBE) reporting guidelines.

### Measures

#### Nighttime Sleep Duration

Parent-reported sleep information was collected at 6, 18, and 30 months, and at 3.5, 4 to 5, 5 to 6, and 6 to 7 years. Nighttime sleep duration was calculated from questions asking what time the child normally went to sleep in the evening and woke up in the morning.

#### Psychotic Outcomes at 24 Years

Psychotic experiences (PEs) were identified through the semistructured Psychosislike Symptom Interview.^27^ PEs occurring in the past 6 months covered the 3 main positive symptom domains: hallucinations, delusions, and thought interference. Cases of PEs were defined as individuals with definite PEs.

We identified individuals with psychotic disorder (PD) at 24 years based on the following criteria^28^: (1) having PEs rated as definite and not associated with sleep or fever, (2) PEs regularly recurring over the previous 6 months, and (3) PEs reported as very distressing or having a very negative impact on social/occupational functioning.

#### Inflammatory Markers at 9 and 15 Years

Blood samples were collected from nonfasting participants during clinic assessment at 9 years at approximately the same time of the day. At 15 years, blood was drawn while fasting at a largely consistent time of day, limiting potential for diurnal effects on inflammatory markers.^29^ Samples were immediately spun, frozen, and stored at −80 °C, and there was no evidence of previous freeze-thaw cycles during storage. High-sensitivity C-reactive protein (CRP) was measured at 9 and 15 years at the same laboratory by automated particle-enhanced immunoturbidimetric assay (Roche UK). There was no evidence of freeze-thaw cycles during storage. Additionally, interleukin 6 (IL-6) at 9 years was measured by single enzyme-linked immunosorbent assay (R&D Systems). All assay coefficients of variation were less than 5%. Higher levels of IL-6^30^ and CRP^31^ were associated with higher probability of infection. IL-6 and CRP levels were log transformed and *z* transformed.^32^

#### Confounders

Multiple family risk factors were assessed using the Family Adversity Index (FAI) during pregnancy and at 2 and 4 years. FAI includes items on early parenthood, housing and family conditions, or social network. Points were summed at each time point for a total FAI score. Early adversity is a well-established risk factor for poor mental health^33^ and poor sleep.^34^

Sleep problems at 24 years were assessed with a self-reported item (“Do you have difficulties getting to sleep or back to sleep?”) from the Revised Clinical Interview Scale,^35^ to control for potential co-occurring sleep problems.

Child sex, gestational age, race and ethnicity, and maternal age when the baby was born were mother reported. Race and ethnicity categories included the following: Bangladeshi, Black African, Black Caribbean, Black Other, Chinese, Indian, Pakistani, White, and other ethnic group (not specified). Further information is available in eAppendix 2 in Supplement 1. These were selected as covariates due to their impact on psychosis and sleep.^36^ The child’s body mass index (BMI) at 9 and 15 years old (ie, same time points as inflammatory markers) was also selected based on the well-known associations between obesity/increased BMI and elevated inflammatory markers.^37,38^

Finally, neurodevelopment disorders, which are also highly linked with both sleep problems^39^ and psychotic symptoms,^40^ were included as covariates. More specifically, we selected ADHD diagnosis at 8 years using the parent-reported Development and Well-Being Assessment,^41^ and autistic spectrum disorder at 9 years, which was measured asking the mother whether the child had a diagnosis of autism, Asperger syndrome, or autism spectrum disorder at the age of 9 years.

### Statistical Analysis

Latent class growth analyses (LCGAs) were performed using Mplus, version 8 (Muthén and Muthén), to identify classes of individuals with differing levels of nighttime sleep duration across childhood. The indicator variables were nighttime sleep duration at 6, 18, and 30 months, and at 3.5, 4 to 5, 5 to 6, and 6 to 7 years. Several models were fit by increasing the number of classes.^42^ The best-fitting classification model was chosen according to fit indices (Bayesian information criteria [BIC] and Vuong-Lo-Mendell-Rubin [VLMR] test).^42^ Lower BIC values suggest better model fit. A significant VLMR value suggests that a K-class model fits the data better than a (K-1) class model. Entropy was also used to select the best model fit; entropy with a value approaching 1 indicates clear delineation of classes. Finally, to decide the optimal class solution, an emphasis was placed on large enough group sizes and clinical relevance. Missing values due to attrition were handled by the full information maximum likelihood estimation method.^42^

We next investigated the prospective associations between the classes identified by LCGA, and psychosis at 24 years, with a particular focus on the class represented by persistently shorter nighttime sleep duration. We conducted logistic regression analyses using SPSS, version 27 (IBM Corp). The derived latent classes from the LCGA were the predictors (with the class with larger sample as reference), and psychosis at 24 years was the outcome. We included the 2 psychotic outcomes (PEs and PD) in separate models. We tested unadjusted associations, as well as adjusted associations controlling for all confounders (except for BMI). To deal with missingness, we conducted logistic regressions to identify significant factors associated with attrition (eTable 1 in Supplement 1). Using the variables associated with selective dropout as the factors, we fitted a logistic regression model to determine weights for each individual using the inverse probability of response.

We last examined the potential role of CRP at 9 and 15 years and IL-6 level at 9 years as mediators in the association between persistent shorter nighttime sleep duration across childhood with psychosis at 24 years. We conducted 6 separate path analyses in SPSS Amos, version 27 (IBM Corp), one per inflammatory maker and psychotic outcome. The independent variable was dichotomized (1 = class referring to persistent shorter nighttime sleep duration; 0 = the other classes). We controlled for FAI, sex, and sleep problems at 24 years, as these were the most relevant covariates as indicated from the logistic regression analyses. We also controlled for BMI at the same time point as the inflammatory marker. We used bootstrapped bias-corrected 95% CIs and *P* values for assessing the significance of the standardized indirect associations. Missing data were dealt with using the full information maximum likelihood method.^43,44^ Data were analyzed from January 30 to August 1, 2023. All *P* values were 2-sided, and *P* < .05 was considered statistically significant.

## Results

Data were available on 3962 young adults (2429 female [61.3%]; 1533 male [38.7%]) with information reported on psychotic outcomes at 24 years (Table 1) and 12 394 children (6254 female [50.5%]; 6140 male [49.5%]). Child race and ethnicity were parent identified as follows: 7 Bangladeshi (0.1%), 11 Black African (0.1%), 76 Black Caribbean (0.6%), 44 Black Other (0.4%), 30 Chinese (0.2%), 54 Indian (0.4%), 22 Pakistani (0.2%), 12 062 White (97.4%), and 82 other (0.7%).

**Table 1.** Descriptive Values of Sociodemographic Variables, Sleep Duration, Inflammatory Markers, and Psychotic Outcomes

Variable	Mean (SD)	No. (%)
Sociodemographics/other covariates		
Sex (boys/girls)		
Female	NA	7348 (48.9)
Male		7691 (51.1)
Race and ethnicity		
Bangladeshi	NA	7 (0.1)
Black African		11 (0.1)
Black Caribbean		76 (0.6)
Black Other		44 (0.4)
Chinese		30 (0.2)
Indian		54 (0.4)
Pakistani		22 (0.2)
White		12 062 (97.4)
Other		82 (0.7)
Birth weight, kg	3.38 (0.58)	NA
FAI, total score	4.38 (4.31)	NA
Maternal age at birth, y	27.99 (4.96)	NA
Gestational age, wk	38.36 (5.51)	NA
ADHD diagnosis at 8 y	NA	175 (2.1)
Autism or Asperger syndrome at 9 y	NA	96 (1.2)
Nighttime sleep duration, h		
6 mo	10.80 (1.36)	NA
18 mo	11.33 (1.08)	NA
30 mo	11.24 (1.01)	NA
3.5 y	11.27 (0.90)	NA
4-5 y	11.39 (0.72)	NA
5-6 y	11.28 (0.72)	NA
6-7 y	10.99 (0.64)	NA
Psychotic outcomes at 24 y		
Psychotic disorders (yes/no)	NA	47/3842 (1.2/98.8)
Psychotic experiences (yes/no)	NA	120/3769 (3.1/96.9)
Inflammatory markers		
CRP at 9 y, mg/L	0.80 (2.88)	NA
CRP at 15 y, mg/L	1.26 (3.97)	NA
IL-6 at 9 y, mg/L	1.29 (1.59)	NA
BMI^a^		
BMI at 9 y	17.72 (2.91)	NA
BMI at 15 y	21.45 (3.57)	NA

Abbreviations: ADHD, attention-deficit/hyperactivity disorder; BMI, body mass index; CRP, C-reactive protein; FAI, Family Adversity Index; IL-6, interleukin-6; NA, not available.

^a^ BMI is calculated as weight in kilograms divided by height in meters squared.

### Latent Classes of Nighttime Sleep Duration

**Figure 1.**
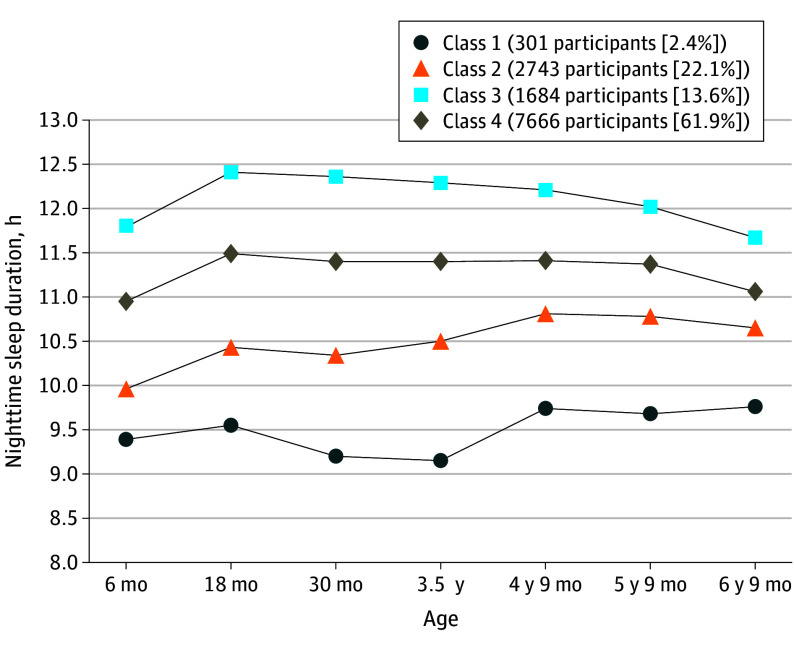
Growth Trajectories of Nighttime Sleep Duration Across Childhood (From 6 Months to 6-7 Years) The latent class growth analyses detected the best model fit for 4 classes. Class 1 represents individuals with persistent shorter sleep duration across time points, which was the main focus of this study. Class 2 refers to individuals with persistent intermediate-shorter sleep duration. Class 3 represents individuals with persistent intermediate-longer sleep duration. Class 4 reflects those individuals with persistent longer sleep duration across time points.

eTable 2 in Supplement 1 shows VLMR, BIC, and entropy for all models assessed (2-6 classes). Overall, a 4-class model provided the best fit. VLMR showed a statistically significant difference for the 2-class, 3-class, and 4-class models (all *P* values <.001). The 5- and 6-class models did not offer a significantly better fit than the 4- and 5-class models, respectively. Decreases in BIC became considerably smaller in 4 classes compared with 3 classes. Finally, the 4-class model reported the highest entropy values (0.799). Further, all classes from the 4-class model included sufficient sample sizes and were clinically relevant. The 4 derived classes are presented in Figure 1. Class 1 represented persistent shorter nighttime sleep duration (301 participants [2.4%]); class 2, persistent intermediate-shorter nighttime sleep duration (2743 participants [21.7%]); class 3, persistent longer nighttime sleep duration (1684 participants [13.6%]); and class 4, persistent intermediate-longer nighttime sleep duration (7666 participants [61.9%]).

### Prospective Associations

The adjusted logistic regressions showed that persistent shorter nighttime sleep duration (class 1) was significantly associated with PD (odds ratio [OR], 2.50; 95% CI, 1.51-4.15; *P* < .001) and PEs (OR, 3.64; 95% CI, 2.23-5.95; *P* < .001), at 24 years (Table 2). Absolute rates of psychosis for each of the nighttime sleep duration classes appear in eTable 3 in Supplement 1.

**Table 2.** Associations between Latent Classes of Nighttime Sleep Duration and Psychotic Disorder and Psychotic Experiences at 24 Years

Psychotic disorder at 24 y	Unadjusted model		Adjusted model	
	OR (95%CI)	*P* value	OR (95% CI)	*P* value
Class 4 (reference)	NA	<.001	NA	.002
Class 3	1.20 (0.32-4.46)	.79	2.49 (0.65-9.53)	.18
Class 2	1.16 (0.74-1.84)	.51	.99 (0.53-1.84)	.98
Class 1^a^	2.49 (1.63-3.80)	<.001	2.50 (1.51-4.15)	<.001
Sex	NA	NA	2.17 (1.34-3.53)	.002
Birth weight, kg			1.93 (1.12-3.14)	.008
Maternal age at birth			0.95 (0.90-0.99)	.03
FAI total score			1.05 (1.01-1.09)	.02
Gestational age			1.16 (0.98-1.39)	.09
Race and ethnicity			1.56 (0.45-5.35)	.48
Sleep problems at 24 y			3.02 (1.91-4.77)	<.001
ADHD at 8 y			0.84 (0.42-1.96)	.48
Autism/Asperger at 9 y			0.95 (0.45-2.13)	.89
**Psychotic experience at 24 y**				
Class 4 (reference)	NA	<.001	NA	<.001
Class 3	1.74 (1.45-2.08)	<.001	1.53 (1.18-1.99)	<.001
Class 2	1.61 (1.29-2.01)	<.001	1.41 (1.13-1.77)	.003
Class 1^a^	2.44 (1.57-3.80)	<.001	3.64 (2.23-5.95)	<.001
Sex	NA	NA	1.01 (0.83-1.22)	.97
Birth weight, kg			1.01 (0.82-1.25)	.90
Maternal age at birth			0.99 (0.97-1.01)	.43
FAI total score			1.05 (1.03-1.07)	<.001
Gestational age			1.07 (0.99-1.14)	.06
Race and ethnicity			0.56 (0.25-1.25)	.16
Sleep problems at 24 y			2.01 (1.67-2.43)	<.001
ADHD at 8 y			0.73 (0.35-1.51)	.40
Autism/Asperger at 9 y			0.99 (0.37-2.66)	>.99

Abbreviations: ADHD, attention-deficit/hyperactivity disorder; FAI, family adversity index; NA, not applicable; OR, odds ratio.

^a^ Class 1 refers to persistent short sleep (and class 4 to the class with higher number of cases).

### Mediating Associations of Inflammatory Markers

**Figure 2.**
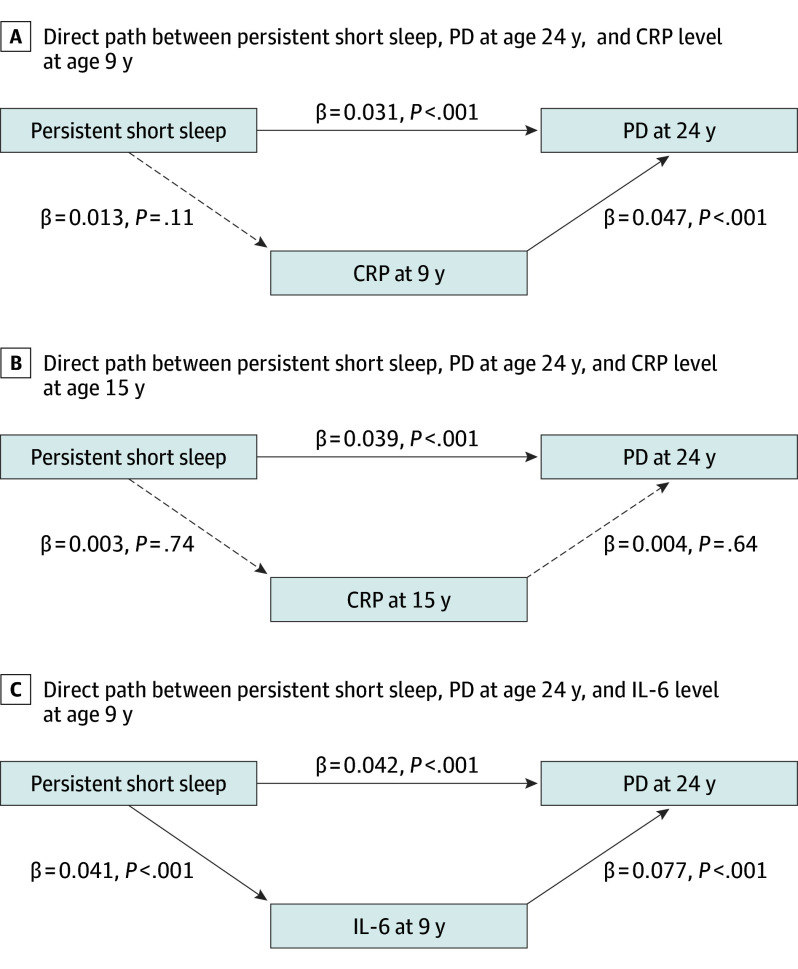
Direct Path Between Persistent Shorter Sleep Across Childhood, Psychotic Disorder (PD) at 24 Years, and Inflammatory Markers Shown are the direct associations of the independent, mediating, and dependent variable. Persistent shorter sleep duration represents the independent variable; C-reactive protein (CRP) at 9 years, CRP at 15 years, and interleukin 6 (IL-6) at 9 years represent the mediating factors, and PD at age 24 years represents the outcome. Three different path analyses were conducted, 1 per inflammatory marker. The covariates also included in these path analyses were Family Adversity Index, sex, and sleep problems at 24 years, as these were the most relevant covariates as indicated from the logistic regression analyses. In addition, we also controlled for body mass index (BMI), ie, BMI at 9 years when IL-6 or CPR at 9 years were included and BMI at 15 years when CRP at 15 years was included. Significant pathways are signified by solid arrows and nonsignificant pathways by dashed arrows.

In examining whether CRP at 9 years partially mediated the association between persistent shorter nighttime sleep duration and PD at 24 years, path analysis model fit indexes indicated good model fit (χ^2^ = 3.11; *P* = .21; root mean square error of approximation [RMSEA] = 0.006; comparative fit index [CIF] = 0.999). However, we did not observe a mediation of CRP at 9 years in the association between exposure and outcome (bias-corrected estimate = 0; 95% CI, −0.001 to 0; *P* = .13). Similar results were obtained for CRP at 15 years as the mediating factor, with a good model (χ^2^ = 1.08; *P* = .78; RMSEA = 0.005; CIF = 0.999) but without an indirect association between exposure and outcome (bias-corrected estimate = 0; 95% CI, −0.001 to 0.001; *P* = .73). When we examined IL-6 level at 9 years as the mediating factor, we observed excellent model fit values (χ^2^ = 1.02; *P* = .80; RMSEA = 0; CIF = 1.000), and also that IL-6 at 9 years partially mediated the association between exposure and outcome (bias-corrected estimate = 0.003; 95% CI, 0.002-0.005; *P* = .007). Direct associations for each of the path analyses conducted for PD appear in Figure 2.

**Figure 3.**
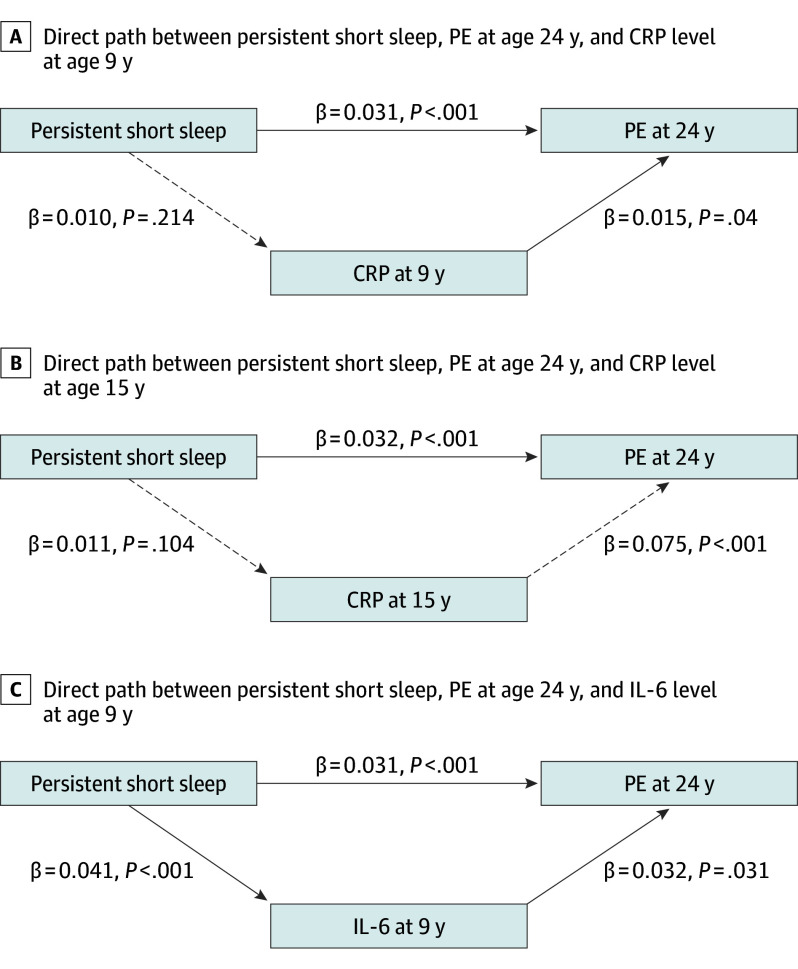
Direct Path Between Persistent Shorter Sleep Across Childhood, Psychotic Experiences (PEs) at 24 Years, and Inflammatory Markers Shown are the direct associations of the independent, mediating, and dependent variable. Persistent shorter sleep duration represents the independent variable; C-reactive protein (CRP) at 9 years, CRP at 15 years, and interleukin 6 (IL-6) at 9 years represent the mediating factors, and PEs at age 24 years represent the outcome. Three different path analyses were conducted, 1 per inflammatory marker. The covariates also included in these path analyses were Family Adversity Index, sex, and sleep problems at 24 years, as these were the most relevant covariates as indicated from the logistic regression analyses. In addition, we also controlled for body mass index (BMI), ie, BMI at 9 years when IL-6 or CPR at 9 years were included, and BMI at 15 years when CRP at 15 years was included. Significant pathways are signified by solid arrows and nonsignificant pathways by dashed arrows.

Regarding PEs at 24 years as the outcome, similar results as the path analyses reported above were obtained (eAppendix 3 in Supplement 1). Notably, and as it happened with PD, we observed excellent model fit values when we examined IL-6 level at 9 years as the mediating factor (χ^2^ = 1.01; *P* = .60; RMSEA = 0; CIF = 1.000), observing also only that IL-6 level at 9 years partially mediated the association between exposure and outcome (bias-corrected estimate = 0.002; 95% CI, 0.001-0.003; *P* = .03). Direct associations for each of the path analyses for PEs appear in Figure 3.

## Discussion

To our knowledge, this is the first study to report that persistent shorter sleep duration across childhood was associated with an increased risk of psychosis in young adulthood. Further, we found that these associations can be partially mediated by specific inflammatory levels (ie, increased levels of IL-6 at 9 years). However, our results indicate that although significant, the size of the association and the proportion of the association mediated was low, which indicates that other factors are also potentially explaining these associations. Future studies should further explore the specific role of inflammation as a potential mediating factor in the prospective associations between sleep and psychosis, together with other potentially relevant mediating factors.

We first found that persistent shorter sleep duration from infancy until childhood was associated with both PEs and PD in young adulthood. Several studies have investigated the predictive role of sleep loss in psychotic symptoms by restricting or depriving sleep in healthy adults, finding that sleep loss leads to increases in psychotic symptoms.^10^ However, these studies are only able to explore the short-term associations of sleep loss on psychosis and not the long-term associations.^10,11^ Previous large epidemiological studies have also shown a correlation between sleep problems and psychotic disturbances.^45,46,47^ However, these studies have largely examined sleep disturbance in adulthood and have been reliant on cross-sectional reports assessing concurrent sleep and psychotic experiences or short-term manipulations of sleep. Although previous prospective studies investigating sleep duration in childhood have not observed an association with psychotic experiences in early and later adolescence,^12,13,14^ these assessed sleep duration at specific time points in childhood rather than the trajectory of sleep duration over time. These prior studies also assessed psychotic experiences in adolescence. Our finding that a trajectory of sleep duration representing persistent lack of sleep in childhood was associated with psychosis at 24 years suggests that chronicity of sleep loss is more important than lack of sleep at a specific time point.^5^ This is likely due to sleep duration being highly variable in childhood,^48^ necessitating multiple assessments over childhood to capture its developmental course. Our findings also may suggest that the detrimental impact of inadequate sleep during childhood does not manifest until young adulthood in association with psychosis, coinciding with the median age of onset for these disorders.^49^ Finally, 2 of the prospective studies mentioned previously,^12,13^ which also used ALSPAC data, found that nightmares at specific time points in childhood, but not sleep duration, were associated with a greater risk of developing psychotic symptoms in adolescence. Together with our findings, this may suggest that persistent shorter sleep across childhood could itself be partly attributed to a higher incidence of nightmares at earlier developmental stages. Therefore, future research should consider the role of other childhood sleep problems in the associations between chronic shorter sleep and psychosis.

We additionally tested for the potential mediating role of inflammation in the prospective associations between persistent shorter sleep duration and psychosis. We found that levels of IL-6, but not CRP, partially mediated these associations. Longitudinal studies in adults have shown that short sleep duration prospectively associates with increases in inflammation, including CRP and IL-6 levels.^20^ Further, existing cross-sectional studies also indicate shorter sleep duration and greater CRP in young adolescents.^50^ Finally, a recent review supports that sleep deprivation alters inflammatory immune processes via multiple pathways, which could lead to increased susceptibility to chronic inflammatory diseases,^51^ supporting potential biological plausibility that persistent lack of sleep over time might lead to inflammatory alterations in the future. Overall, these findings are potentially explained by the dynamic role of sleep in regulating the immune system,^17^ and thus, lack of sleep may lead to changes in the effector systems that regulate the immune system and consequently lead to abnormal increases in inflammatory responses. However, although the existent evidence supports that inflammation is a consequence of persistent sleep disturbance,^20^ a risk factor for psychosis^32^ and a potential mediating factor between sleep and psychosis,^16^ to date, only 1 study^21^ has investigated the co-occurrence of sleep disturbance and inflammation in outpatients with schizophrenia. However, as this was a cross-sectional study, the authors were unable to identify whether inflammatory markers mediated the sleep problems in these patients with schizophrenia.

The findings of our current study are congruent with a recent study of ours using ALSPAC data where we found that IL-6 level, but not CRP level (both at 9 years), partially mediated the associations between sleep problems in childhood and ADHD diagnosis at 10 years.^2^ Another recent study^52^ from our group also using ALSPAC data found that CRP level at 9 and 15 years, but not IL-6 level at 9 years, partially mediated the associations between persistent anxiety across childhood and psychosis at 24 years. Based on this limited evidence, we could hypothesize that different physiological actions associate with each of these inflammatory markers^53^; thus, IL-6 level may be more sensitive to sleep problems, although anxiety may have a greater association with CRP levels, in the pathway to psychosis. A role for an IL-6-specific pathway in early developmental processes is also supported by a recent mendelian randomization study in the UK Biobank, which found that genetically predicted IL-6 level, but not CRP level, was associated with brain structure,^54^ suggesting that early exposure to elevated IL-6 levels may affect development of brain structure in areas relevant to neurodevelopment (and therefore sleep), whereas CRP level might be more environmentally responsive.

### Strengths and Limitations

Strengths of our study include the large population-based sample size, the longitudinal design, and the inclusion of sleep duration across childhood. However, this study has some limitations. First, this study only focused on parent- or self-reported sleep, which could be different from objective sleep.^55^ For instance, parent- or self-reported sleep is subject to bias and subjective interpretation, in addition to be influenced by internal and/or external factors (eg, parents who are under greater stress or themselves are sleeping poorly might interpret their child as sleeping less). Second, daytime sleep duration was not available for all the time points, and thus, we were not able to provide trajectories on daytime or total sleep duration. Third, most participants were of White race and were all residents in the same geographic area in the UK, which limits the generalizability of our findings. Fourth, our results could be explained by other potential underlying factors that have not been explored in this study. Examples of these include common genetic factors or existing anxiety symptoms or those that have yet to be defined and/or evaluated. We focused on inflammation as it is modifiable and due to its links with both sleep and psychosis. The focus of future research should be to untangle the contribution of other relevant mediating factors. Fifth, in the absence of an experimental manipulation or intervention, we cannot imply here that shorter sleep duration is causally contributing to later psychotic experiences, and it is possible that the association might be due to other factors associated with both childhood sleep and psychosis. Although we have controlled for several relevant factors in these associations, other unknown factors might potentially explain these associations. Finally, although here we focused on the associations with psychosis, it is likely that these associations are transdiagnostic considering that inflammatory markers have been associated with a range of psychiatric disorders in this same cohort.^2,32,52^ Therefore, future studies should explore the pathways of associations with other mental health outcomes.

## Conclusions

Results of this cohort study suggest that persistent shorter sleep duration across childhood was associated with higher risk of developing psychosis in young adulthood. Further, IL-6 level, but not CRP level, partially mediated these prospective associations. Our findings highlight the necessity of addressing short sleep duration in children, as persistence of these sleep problems may be a risk factor for subsequent psychosis. Our study also provides evidence to develop future targeted early interventions in children addressing both sleep duration and specific inflammatory levels, to prevent future adverse outcomes.

## Notes

Supplement 1. **eAppendix 1.** Details of the ALSPAC Cohort **eAppendix 2.** Details About Ethnicity **eTable 1.** Differences in Sociodemographic Variables Between Nonparticipating and Participating Individuals in the Study **eTable 2.** Bayesian Information Criterion, Vuong-Lo-Mendell-Rubin Likelihood Test *P* Values, and Entropy for Classes 2 to 6, for Each of the Nighttime Sleep Duration Values **eTable 3.** Absolute Rates of Psychosis for Each of the Nighttime Sleep Duration Classes **eAppendix 3.** Mediating Effect of Inflammatory Markers in the Association Between Persistent Shorter Nighttime Sleep Duration and PEs at 24 Years

Supplement 2.
Data Sharing Statement.
