# Supplementary material for: Role of Inflammation in Short Sleep Duration Across Childhood and Psychosis in Young Adulthood
Source: JAMA Psychiatry. 2024 May 8;81(8):825–33. doi: 10.1001/jamapsychiatry.2024.0796 (PMC11079792; doi:10.1001/jamapsychiatry.2024.0796)
Supplement: Supplement 1. — eAppendix 1. Details of the ALSPAC Cohort eAppendix 2. Details About Ethnicity eTable 1. Differences in Sociodemographic Variables Between Nonparticipating and Participating Individuals in the Study eTable 2. Bayesian Information Criterion, Vuong-Lo-Mendell-Rubin Likelihood Test P Values, and Entropy for Classes 2 to 6, for Each of the Nighttime Sleep Duration Values eTable 3. Absolute Rates of Psychosis for Each of the Nighttime Sleep Duration Classes eAppendix 3. Mediating Effect of Inflammatory Markers in the Association Between Persistent Shorter Nighttime Sleep Duration and PEs at 24 Years [file jamapsychiatry-e240796-s001.pdf]

Supplementary Online Content

Morales-Muñoz I, Marwaha S, Upthegrove R, Cropley V. Role of inflammation in short sleep duration across childhood and psychosis in young adulthood. *JAMA Psychiatry*. Published online May 8, 2024. doi:10.1001/jamapsychiatry.2024.0796

eAppendix 1. Details of the ALSPAC Cohort

eAppendix 2. Details About Ethnicity

eTable 1. Differences in Sociodemographic Variables Between Nonparticipating and Participating Individuals in the Study

eTable 2. Bayesian Information Criterion, Vuong-Lo-Mendell-Rubin Likelihood Test *P* Values, and Entropy for Classes 2 to 6, for Each of the Nighttime Sleep Duration Values

eTable 3. Absolute Rates of Psychosis for Each of the Nighttime Sleep Duration Classes

eAppendix 3. Mediating Effect of Inflammatory Markers in the Association Between Persistent Shorter Nighttime Sleep Duration and PEs at 24 Years

This supplementary material has been provided by the authors to give readers additional information about their work.

© 2024 Morales-Muñoz I et al. *JAMA Psychiatry.*

eAppendix 1. Details of the ALSPAC Cohort

The initial number of pregnancies enrolled was 14,541 (for these at least one questionnaire was returned, or a "Children in Focus" clinic had been attended by 19/07/99). Of these initial pregnancies, there was a total of 14676 foetuses, resulting in 14062 live births and 13988 children who were alive at 1 year of age. When the oldest children were approximately 7 years of age, an attempt was made to bolster the initial sample with eligible cases who had failed to join the study originally. As a result, in our study, as some variables were collected from the age of seven onwards there were data available for more than the 14541 pregnancies mentioned above. Informed consent for the use of data collected via questionnaires and clinics was obtained from participants following the recommendations of the ALSPAC Ethics and Law Committee at the time. Ethical approval was obtained from the ALSPAC Law and Ethics committee and the local research ethics committees.

© 2024 Morales-Muñoz I et al. *JAMA Psychiatry.*

eAppendix 2. Details About Ethnicity

The ethnicity of the child was reported by the mother, and this variable was requested as part of the socio-demographic variables requested by the ALSPAC cohort. The categories of ethnicity provided by the ALSPAC and the number of participants (and %) for each category in our study were: White (N=12,062; 97.4%), Black Caribbean (N=76, 0.6%), Black African (N=11, 0.1%), Other Black (N=44, 0.4%), Indian (N=54, 0.4%), Pakistani (N=22, 0.2%), Bangladeshi (N=7, 0.1%), Chinese (N=30, 0.2%), Other (N=82, N=0.7%). For the purpose of our study, and considering that ethnicity was included as a covariate and that our sample was predominently of White ethnicity, we decided to create to categories of White and Non-White. For Non-White category we indlude all the categories above that did not comprise the White category.

© 2024 Morales-Muñoz I et al. *JAMA Psychiatry.*

eTable 1. Differences in sociodemographic variables between nonparticipating and participating individuals in the study

	Non-participating group in the study		Participating group in the study (at 24 years old)		Non-participating versus participating	
	Mean	*SD*	*Mean*	*SD*	*OR (95% CI)*	*p*
Maternal age when born	27.49	4.99	29.45	4.56	1.08 (1.08, 1.09)	<0.001
Gestational age	38.00	6.22	39.49	1.80	1.09 (1.08, 1.11)	<0.001
Birth weight, grams	3371.23	596.18	3410.33	532.74	1.00 (1.00, 1.00)	0.001
Family Adversity score	4.74	4.47	3.61	3.84	0.936 (0.93, 0.95)	<0.001
	**Non-participating group in the study**		**Participating group in the study**			
	N	*%*	*N*	*%*		
Sex						
Male / Female	6233 / 4919	55.9 / 44.1	1458 / 2429	37.5 / 62.5	0.47 (0.44, 0.51)	<0.001
Ethnicity						
White / Other	8657 / 248	97.2 / 2.8	3405 / 78	97.8 / 2.2	1.251 (0.97, 1.62)	0.089

The individuals associated with attrition at 24 years were more often boys, their mothers were younger when baby was born, the gestational age was shorter, they weighed less at birth, and they had higher socioeconomic levels.

© 2024 Morales-Muñoz I et al. *JAMA Psychiatry*.

eTable 2. Bayesian Information Criterion, Vuong-Lo-Mendell-Rubin Likelihood Test P Values and Entropy for classes 2-6, for each of the nighttime sleep duration values

Nighttime sleep duration from 6mo to 7y	BIC	VLMR-P	Entropy
2 classes	173960.109	<0.001	0.750
3 classes	171052.404	<0.001	0.769
4 classes	170000.824	<0.001	0.788
5 classes	169365.553	0.2324	0.683
6 classes	169324.312	0.3321	0.711

BIC=Bayesian information criterion; VLMR-P= Vuong-Lo-Mendell-Rubin likelihood ratio test.

eTable 3. Absolute rates of psychosis for each of the nighttime sleep duration classes

	Psychotic disorder at 24 years			
	Yes		No	
	**N**	**%**	**N**	**%**
**Class 1**	4	4.3	63	95.7
**Class 2**	11	1.4	791	98.6
**Class 3**	10	2.1	475	97.9
**Class 4**	20	0.9	2154	99.1
	**Psychotic experiences at 24 years**			
	**Yes**		**No**	
	**N**	**%**	**N**	**%**
**Class 1**	7	10.4	60	89.6
**Class 2**	68	8.5	734	91.5
**Class 3**	38	7.8	447	92.2
**Class 4**	116	5.3	2058	94.7

Class 1=persistent shorter night-time sleep duration; Class 2=persistent intermediate-shorter night-time sleep duration; Class 3=persistent longer night-time sleep duration; Class 4=persistent intermediate-longer night-time sleep duration.

© 2024 Morales-Muñoz I et al. *JAMA Psychiatry.*

eAppendix 3. Mediating effect of inflammatory markers in the association between persistent shorter night-time sleep duration and PEs at 24 years

Regarding PEs at 24 years as the outcome, the path analyses model fit indexes when CRP at 9 years was included as mediator indicated a good model fit ($\chi 2$=3.41, P=.16; RMSEA=0.007; CIF=0.996). However, we did not observe an indirect effect of CRP at 9 years in the association between exposure and outcome (bias-corrected estimate, 0.001; 95% CI=-0.001 to 0.001, p=0.165). We also obtained a good model fit when we included CRP at 15 years as the mediating factor ($\chi 2$=1.47, P=.33; RMSEA=0.008; CIF=0.989), but also without an indirect effects of CRP at 15 years (bias-corrected estimate, -0.001; 95% CI=-0.002 to 0.001, p=0.173). Similar to what happened with PD at 24 years, we observed excellent model fit values when we examined IL-6 at 9 years as the mediating factor between persistent short sleep and PEs at 24 years ($\chi 2$=1.01, P=0.60; RMSEA=0.000; CIF=1.000), observing also only a partially mediating effect of IL-6 at 9 years in the association between exposure and outcome (bias-corrected estimate=0.002; 95% CI=0.001 to 0.003, p=0.028).

© 2024 Morales-Muñoz I et al. *JAMA Psychiatry.*
