# Supplementary material for: Role of Inflammation in Short Sleep Duration Across Childhood and Psychosis in Young Adulthood
Source: JAMA Psychiatry. 2024 May 8;81(8):825–33. doi: 10.1001/jamapsychiatry.2024.0796 (PMC11079792; doi:10.1001/jamapsychiatry.2024.0796)
Supplement: Supplement 2. — Data Sharing Statement. [file jamapsychiatry-e240796-s002.pdf]

Data Sharing Statement

Morales-Muñoz. Role of Inflammation in Short Sleep Duration Across Childhood and Psychosis in Young Adulthood. *JAMA Psychiatry*. Published May 08, 2024.
doi:10.1001/jamapsychiatry.2024.0796

Data
Data available: No

Additional Information
Explanation for why data not available: This study used the ALSPAC cohort study, and due to the ALSPAC policy, researchers should request data and get access to the data through the ALSPAC data sharing policy. Once researchers get approval to get access to the data by ALSPAC, then they will be able to get access to the data that we have used.
